# Supplementary material for: Mathematical modelling of the role of GADD45β in the pathogenesis of multiple myeloma
Source: R Soc Open Sci. 2020 May 13;7(5):192152. doi: 10.1098/rsos.192152 (PMC7277253; doi:10.1098/rsos.192152)
Supplement: APPENDIX.pdf [file rsos192152supp1.pdf]

Appendix A

A1 Figure 1 in the model developed by Ji et al. (2014)

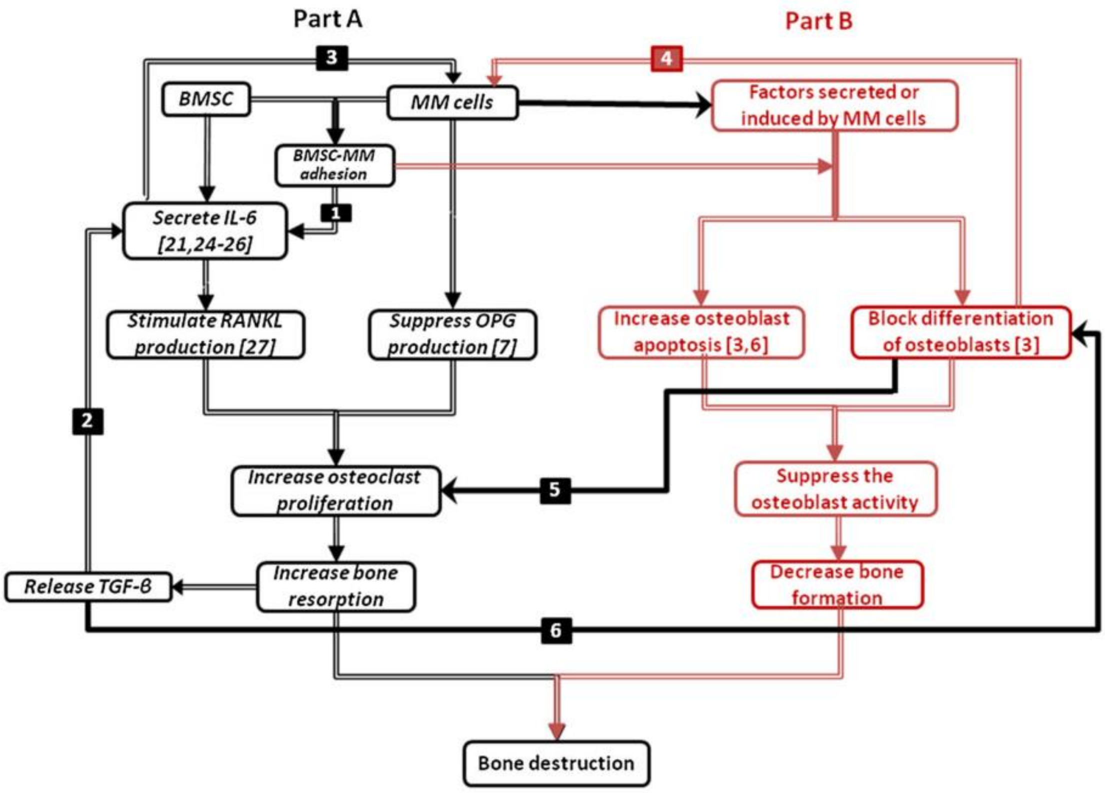

Figure A1. Proposed cellular interaction in MM development.

A2 Tables of π functions and definitions of the concentrations in the model developed by Ji et al. (2014)

Table A2.1: Definitions of the π functions used in the model.

$TGF\beta$ stimulates the differentiation of OB_u into OB_p	$\pi_{act,OB_u}^{TGF\beta} = \frac{TGF\beta}{K_{D1,TGF\beta} + TGF\beta}$
$TGF\beta$ inhibits the differentiation of OB_p into OB_a	$\pi_{rep,OB_p}^{TGF\beta} = \frac{1}{1 + (TGF\beta / K_{D2,TGF\beta})}$
BMSC-MM cell adhesion	$\pi_{rep,OB_p}^{VCAM1} = \frac{1}{1 + (VCAM1 / K_{D,VCAM1,OB_p,rep})}$

BMSC-MM cell adhesion stimulating the apoptosis of osteoblasts	$\pi_{act,OB_a}^{VCAM1} = \frac{VCAM1}{VCAM1 + K_{D,VCAM1,OB_a,act}}$
the effect of TGF-β and RANKL on osteoclastic and osteoblastic lineages	$\pi_{act,OC_P}^{RANKL} = \frac{RANKL}{K_{D,RANKL} + RANKL}$
$TGF\beta$ promotes the apoptosis of OB_a	$\pi_{act,OC_a}^{TGF\beta} = \frac{TGF\beta}{K_{D3,TGF\beta} + TGF\beta}$
IL-6 regulation of the proliferation of MM cells	$\pi_{act,MM}^{IL6} = \frac{IL6}{IL6 + K_{D,IL6,MM,act}}$
the effect of MM-BMSC on the proliferation of MM cells	$\pi_{act,MM}^{VCAM1} = \frac{VCAM1}{VCAM1 + K_{D,VCAM1,MM,act}}$
SLRPs produced by mature osteoblasts suppressing the proliferation of MM cells	$\pi_{rep,MM}^{SLRPs} = \frac{1}{1 + (SLRPs/K_{D,SLRPs,MM,rep})}$
PTH stimulates the production of RANKL	$\pi_{act,RANKL}^{PTH} = \frac{PTH}{K_{D1,PTH} + PTH}$
PTH inhibits the production of OPG	$\pi_{rep,OPG}^{PTH} = \frac{1}{1 + (PTH/K_{D2,PTH})}$
IL-6 stimulates the production of RANKL	$\pi_{act,RANKL}^{IL6} = \frac{IL6}{IL6 + K_{D,IL6,RANKL,act}}$
VAL-4 stimulates the production of IL-6	$\pi_{act,IL6}^{VLA4} = \frac{VLA4}{VLA4 + K_{D,VLA4,IL6,act}}$
TGF?β stimulates the production of IL-6	$\pi_{act,IL6}^{TGF\beta} = \frac{TGF\beta}{TGF\beta + K_{D,TGF\beta,IL6,act}}$

Table A2.2: Definitions of the concentrations of RANKL, OPG, TGF-β, PTH, IL-6, SLRPs, VLA-4 and VCAM -1.

$RANKL$	$\frac{P_{RANKL,d}+\beta_{RANKL}\cdot OB_p}{(1+K_{A,OPG}\cdot OPG+K_{A,RANK}\cdot RANK)\cdot(\frac{\beta_{RANKL}}{R^{RANKL}\cdot\pi_{act,RANKL}^{IL6}\cdot\pi_{act,RANKL}^{PTH}}+D_{RANKL})}$
OPG	$\frac{P_{OPG,d}+\beta_{OPG}\cdot OB_a\cdot\pi_{rep,OPG}^{PTH}}{\left(\frac{\beta_{OPG}\cdot OB_a\cdot\pi_{rep,OPG}^{PTH}}{OPG_{max}}+D_{OPG}+D_{OPG,MM}\cdot MM\right)}$
$TGF\beta$	$\frac{\alpha\cdot K_{res}\cdot OC_a+S_{TGF\beta}}{\widetilde{D}_{TGF\beta}}$
PTH	$\frac{\beta_{PTH}+P_{PTH,d}}{\widetilde{D}_{PTH}}$
$IL6$	$\frac{P_{IL6,d}+\beta_{IL6}\cdot OB_u\cdot\pi_{act,IL6}^{TGF}\cdot\pi_{act,IL6}^{VLA4}}{\left(\frac{\beta_{IL6}\cdot OB_u\cdot\pi_{act,IL6}^{TGF}\cdot\pi_{act,IL6}^{VLA4}}{IL6_{max}}+D_{IL6}\right)}$
$SLRPs$	$\frac{\beta_{SLRPs}\cdot OB_a+P_{SLRPs,d}}{\left(\frac{\beta_{SLRPs}\cdot OB_a}{SLRPs_{max}}+\widetilde{D}_{SLRPs}\right)}$
$VLA4$	$\frac{P_{VLA4,d}+\beta_{VLA4}\cdot MM}{(1+K_{A,VCAM1}\cdot VCAM1_{tot})\cdot(\frac{\beta_{VLA4}}{R^{VLA4}}+D_{VLA4})}$
$VCAM1$	$\frac{VCAM1_{tot}}{1+K_{A,VCAM1}+VLA4}$

The definitions and values for parameters in above equations can be found in Ji et al. (2014).

Appendix B

B1 Mathematical model of JNK signalling pathway

$$\frac{d\ MAPKKK}{dt} = v_2 - v_1 \quad \text{(B1)}$$

$$\frac{d\ MAPKKK\_P}{dt} = v_1 - v_2 \quad \text{(B2)}$$

$$\frac{d\ MKK7}{dt} = v_6 - v_3 \quad \text{(B3)}$$

$$\frac{d\ MKK7\_P}{dt} = v_3 + v_5 - v_4 - v_6 \quad \text{(B4)}$$

$$\frac{d\ MKK7\_PP}{dt} = v_4 - v_5 \quad \text{(B5)}$$

$$\frac{d\ JNK}{dt} = v_{10} - v_7 \quad \text{(B6)}$$

$$\frac{d\ JNK\_P}{dt} = v_7 + v_9 - v_8 - v_{10} \quad \text{(B7)}$$

$$\frac{d\ JNK\_PP}{dt} = v_8 - v_9 \quad \text{(B8)}$$

$$MAPKKK = k_{MAPKKK} \cdot MM - d_{MAPKKK} \cdot MAPKKK \quad \text{(B9)}$$

$$MKK7 = k_{MKK7} \cdot MM - d_{MKK7} \cdot MKK7 - (k_{GM} \cdot GADD45\beta \cdot MKK7 - d_{GM} \cdot (GADD45\beta|MKK7)) \quad \text{(B10)}$$

$$JNK = k_{JNK} \cdot MM - d_{JNK} \cdot JNK \quad \text{(B11)}$$

Table B1: Definitions of v_1 to v_{10} used in equations B1 to B8.

$v_1 = \dfrac{V_1 \cdot MAPKKK}{K_1 + MAPKKK}$	$v_6 = \dfrac{V_6 \cdot MKK7\_P}{K_6 + MKK7\_P}$
$v_2 = \dfrac{V_2 \cdot MAPKKK\_P}{K_2 + MAPKKK\_P}$	$v_7 = \dfrac{k_7 \cdot MKK7\_PP \cdot JNK}{K_7 + JNK}$
$v_3 = \dfrac{k_3 \cdot MAPKKK\_P \cdot MKK7}{K_3 + MKK7}$	$v_8 = \dfrac{k_8 \cdot MKK7\_PP \cdot JNK\_P}{K_8 + JNK\_P}$
$v_4 = \dfrac{k_4 \cdot MAPKKK \cdot MKK7\_P}{K_4 + MKK7\_P}$	$v_9 = \dfrac{V_9 \cdot JNK\_PP}{K_9 + JNK\_PP}$
$v_5 = \dfrac{V_5 \cdot MKK7\_PP}{K_5 + MKK7\_PP}$	$v_{10} = \dfrac{V_{10} \cdot JNK\_P}{K_{10} + JNK\_P}$

The definitions and values for parameters in above equations can be found in Boris N. Kholodenko (2000).

Appendix C

C1 Mathematical model of NF-κB signalling pathway

$$\frac{d}{dt}IKKn(t) = k_{prod} - k_{deg}IKKn(t) - T_R k_1 IKKn(t) \quad \text{(C1)}$$

$$\frac{d}{dt}IKKa(t) = T_R k_1 IKKn(t) - k_3 IKKa(t) - T_R k_2 IKKa(t) \cdot A20(t) - k_{deg}IKKa(t)$$
$$-a_2 IKKa(t) \cdot I\kappa B\alpha(\mathrm{t}) + t_1(IKKa|I\kappa B\alpha)(\mathrm{t})$$
$$-a_3 IKKa(t) \cdot (I\kappa B\alpha|NF\kappa B)(t)$$
$$+t_2(IKKa|I\kappa B\alpha|NF\kappa B)(t) \quad \text{(C2)}$$

$$\frac{d}{dt}IKKi(t) = k_3 IKKa(t) + T_R k_2 IKKa(t) \cdot A20(t) - k_{deg}IKKi(t) \quad \text{(C3)}$$

$$\frac{d}{dt}(IKKa|I\kappa B\alpha)(t) = a_2 IKKa(t) \cdot I\kappa B\alpha(t) - t_1(IKKa|I\kappa B\alpha)(t) \quad \text{(C4)}$$

$$\frac{d}{dt}(IKKa|I\kappa B\alpha|NF\kappa B)(t) = a_3 IKKa(t) \cdot (I\kappa B\alpha|NF\kappa B)(t)$$
$$-t_2(IKKa|I\kappa B\alpha|NF\kappa B)(t) \quad \text{(C5)}$$

$$\frac{d}{dt}NF\kappa B(t) = c_{6a}(I\kappa B\alpha|NF\kappa B)(t) - a_1 NF\kappa B(t) \cdot I\kappa B\alpha(t)$$
$$+t_2(IKKa|I\kappa B\alpha|NF\kappa B)(\mathrm{t}) - i_1 NF\kappa B(t) \quad \text{(C6)}$$

$$\frac{d}{dt}NF\kappa B_n(t) = i_1 k_v NF\kappa B(t) - a_1 I\kappa B\alpha_n(t) \cdot NF\kappa B_n(t) \quad \text{(C7)}$$

$$\frac{d}{dt}A20(t) = c_4 A20_t(t) - c_5 A20(t) \quad \text{(C8)}$$

$$\frac{d}{dt}A20_t(t) = c_2 + c_1 NF\kappa B_n(t) - c_3 A20_t(t) \quad \text{(C9)}$$

$$\frac{d}{dt}I\kappa B\alpha(t) = -a_2 IKKa(t) \cdot I\kappa B\alpha(t) - a_1 I\kappa B\alpha(t) \cdot NF\kappa B(t) + c_{4a} I\kappa B\alpha_t(t)$$
$$-c_{5a} I\kappa B\alpha(t) - i_{1a} I\kappa B\alpha(t) + e_{1a} I\kappa B\alpha_n(\mathrm{t}) \quad \text{(C10)}$$

$$\frac{d}{dt}I\kappa B\alpha_n(t) = -a_1 I\kappa B\alpha_n(t) \cdot NF\kappa B_n(t) + i_{1a} k_v I\kappa B\alpha(t) - e_{1a} k_v I\kappa B\alpha_n(t) \quad \text{(C11)}$$

$$\frac{d}{dt}I\kappa B\alpha_t(t) = c_{2a} + c_{1a} NF\kappa B_n(t) - c_{3a} I\kappa B\alpha_t(t) \quad \text{(C12)}$$

$$\frac{d}{dt}(I\kappa B\alpha|NF\kappa B)(t) = a_1 I\kappa B\alpha(t) \cdot NF\kappa B(t) - c_{6a}(I\kappa B\alpha|NF\kappa B)(t)$$
$$-a_3 IKKa(t) \cdot (I\kappa B\alpha|NF\kappa B)(t)$$
$$+e_{2a}(I\kappa B\alpha_n|NF\kappa B_n)(\mathrm{t}) \quad \text{(C13)}$$

$$\frac{d}{dt}(I\kappa B\alpha_n|NF\kappa B_n)(t) = a_1 I\kappa B\alpha_n(t) \cdot NF\kappa B_n(t) - e_{2a} k_v (I\kappa B\alpha_n|NF\kappa B_n)(t) \quad \text{(C14)}$$

$$\frac{d}{dt}GADD45\beta_t(t) = c_{2c} + c_{1c} NF\kappa B_n(t) - c_{3c} GADD45\beta_t(t) \quad \text{(C15)}$$

$$\frac{d}{dt}GADD45\beta = k_G \cdot GADD45\beta_t - d_G \cdot GADD45\beta - (k_{GM} \cdot GADD45\beta \cdot MKK7$$
$$-d_{GM} \cdot (GADD45\beta|MKK7)) \quad \text{(C16)}$$

The definitions and values for parameters in above equations can be found in Lipniacki et al. (2004).
